# A reproducible pipeline for activity-based travel demand generation in England

**DOI:** 10.1177/23998083251379620

**Published:** 2025-09-19

**Authors:** Hussein Mahfouz, Sam F. Greenbury, Bowen Zhang, Stuart Lynn, Tao Cheng

**Affiliations:** 1 Institute for Transport Studies, 4468 University of Leeds, Leeds, UK; 2 The Alan Turing Institute, London, UK; 3 Department of Civil, Environmental & Geomatic Engineering, 4919 University College London, London, UK

**Keywords:** activity-based travel demand, synthetic populations, open-source, reproducible

## Abstract

Agent-based transport models are gaining popularity due to their ability to model features such as heterogenous individual behaviour, household dependencies, and new dynamic modes of travel. Such models require as input disaggregate population datasets with detailed daily activity diaries (activity-based travel demand). While there is extensive literature on activity-based travel demand generation, few open-source tools are available for producing such datasets, and those that do exist are often difficult to adapt to different study areas. In this work, we present an open-source modular pipeline for generating activity-based travel demand for any region in England, producing individuals with household structures and geographically and temporally explicit daily activity plans. The framework includes activity scheduling and location assignment for a synthetic population, as well as self-consistency and validation frameworks to help fine-tune parameters.

## Introduction

Data availability, computational resources, and the desire to capture individual-level impacts of policy decisions have led to an increase in adoption of agent-based transport models (AgBMs) (Bastarianto et al., 2023; Kagho et al., 2020).

To realistically model travel behaviour, AgBMs require representative synthetic populations with detailed daily activity schedules and locations (activity-based travel demand). The generation of these datasets, however, represents a significant bottleneck that hinders reproducible science and the wider adoption of AgBMs in practice. To our knowledge, the only open-source pipeline for activity generation and location assignment is Hörl and Balac (2021), which is designed for France and requires familiarity with the codebase to adapt for use in other regions.

To address this challenge, this paper introduces an open-source pipeline for generating activity-based travel demand for any region in England. It enriches the static populations from the Synthetic Population Catalyst (SPC) (Salat et al., 2023) with travel demand attributes. While the SPC provides realistic individuals with household structures, sociodemographic attributes, and home locations, our pipeline enriches this population with daily activity plans. This includes, for each individual, what activities they undertake, when these activities occur, the spatial location of the activities, and the travel modes used. The primary contribution is therefore a piece of scholarly infrastructure (as defined in Arribas-Bel et al. (2021)): a reproducible pipeline that generates outputs compatible with downstream AgBM platforms like MATSim (Horni et al., 2016), lowering the barrier for AgBM and enabling researchers to focus more of their time on policy analysis and simulation.

In the following sections, we present some background on each part of our pipeline, along with alternative methods that could be used (Section 2). We then present the details of our methods (Section 3), and the validation metrics we used throughout (Section 4).

## Background and design rationale

Activity-based travel demand datasets are made up of (a) synthetic populations, with (b) daily activities that are (c) assigned to locations. Below we give an overview of these building blocks, the different methods used for each one, and justify our choice of methods.

### Synthetic populations

Synthetic populations are comprised of individuals and households that are artificial but whose aggregate statistics and properties aim to be representative of a real counterpart population. They provide feature-rich population data through combining multiple data sources and can include granular detail that may not be available regarding their real counterpart. Synthetic populations have been applied in many fields including health (Wu et al., 2022), transport (Lovelace et al., 2014), land use (Lomax et al., 2022), and COVID modelling (Spooner et al., 2021). A wide-range of methodologies can be applied in their construction broadly including synthetic reconstruction, combinatorial optimization, and statistical learning (Fabrice Yaméogo et al., 2021).

Our work uses the synthetic individuals and households from the SPC (Salat et al., 2023) as its foundational population. However, the SPC’s native activity-generation component is unsuitable for creating the detailed daily diaries required for activity-based transport modelling. Specifically, while the SPC assigns individuals to primary destination zones, its activity data has several limitations for this purpose: it does not sequence movements into coherent, multi-stop trip chains; it includes no information on the timing or duration of activities; and it does not specify the mode of travel. For this reason, our work does not use or extend the SPC’s activity-generation module. Instead, we present an entirely new pipeline that takes the static population from the SPC as an input and generates the dynamic, fully specified activity schedules – complete with sequencing, timing, and travel mode – required by downstream simulation platforms like MATSim.

### Generating activity schedules

A number of approaches exist for generating individual activity schedules. *Statistical matching* approaches (D’Orazio et al., 2006) are used to match individuals in a synthetic population to respondents in a travel survey, with the matching being done on demographic and socioeconomic attributes. Unconstrained approaches are common (Hörl and Balac, 2021; Namazi-Rad et al., 2017) and can be implemented even when the travel survey has a small sample size, is not representative of all demographic categories, or does not include enough diversity to have exact matches for all individuals in the synthetic population. *Bayesian networks* have recently been used as an alternative approach to generating activity patterns (De Waal and Joubert, 2022; Joubert and De Waal, 2020; Sallard and Balać, 2023), as their graphical structure can effectively communicate dependencies between attributes and create unobserved activity sequences (Sallard and Balać, 2023). *Deep generative models* have also been applied to activity pattern generation (Koushik et al., 2023; Shone and Hillel, 2025).

While each approach has merits, we selected statistical matching for this pipeline due to its balance of performance, maturity, and ability to handle household-level constraints. Studies comparing Bayesian networks with statistical matching have found that both methods are suitable for replicating a given distribution of activity chains (Sallard and Balać, 2023). Meanwhile, state-of-the-art deep learning approaches are currently focused on generating individual-level schedules, and the incorporation of household interactions and constraints remains an area for future research. Given our requirement to maintain household trip dependencies from the NTS, the well-established statistical matching method remains a practical choice.

### Location assignment

Location assignment refers to assigning individual activities to feasible geographic locations. This includes primary and secondary activities. The former includes trips to fixed locations such as work or school, whereas the latter are trips that fill gaps between primary locations (Hörl and Axhausen, 2023).

For primary location assignment, traditional aggregate models like gravity (Voorhees, 2013), entropy (Wilson, 2013), or radiation (Simini et al., 2012) models are well-studied for estimating flows between zones. However, these are insufficient for agent-based models because they lack individual-level constraints. Specifically, synthetic populations often have detailed information for each agent, such as expected travel times derived from survey data, which must be respected in the location assignment. To address this, Hörl and Balac (2021) combine aggregate data with agent-level detail, using census commuting data to ensure realism at the zonal level, while constraining the final assignment of a specific location based on each individual’s expected travel time or distance from their activity schedule. Our pipeline adopts the same approach to satisfy both zonal and individual-level constraints.

On their own, the approaches for primary location assignment cannot be used to model an entire trip chain which includes secondary (discretionary) locations. Secondary location assignment has been studied through the framework of space-time prisms (Hägerstrand, 1970). Most approaches involve the creation of a choice set for each activity (based on reported travel time, mode used, and activity purpose) and then sampling a location from that choice set. Methods for choosing a location include choice models (Justen et al., 2013; Yoon et al., 2012) and Bayesian networks (Ma and Klein, 2018). As an alternative to these often data-intensive techniques, Hörl and Axhausen (2023) propose an algorithm that uses primary activity locations as anchors to generate realistic secondary location patterns that reproduce the distance distributions in the reference data. This method was selected for our pipeline because its minimal data requirements and avoidance of complex model estimation align with our goal of creating an accessible and reproducible tool that provides preliminary results that can be refined in agent-based simulations.

## Data sources and methods

The methodological approach for this pipeline prioritizes the integration of established, well-understood techniques over the development or inclusion of more novel ones. The primary goal is to provide a transparent and reproducible pipeline that uses familiar methods. The specific methods chosen for each module, such as statistical matching for activity generation and constrained optimization for primary location assignment, were selected for their proven efficacy and relatively straightforward data requirements. The following sections detail the specific implementation of each component.

### Datasets

**Figure 1.**
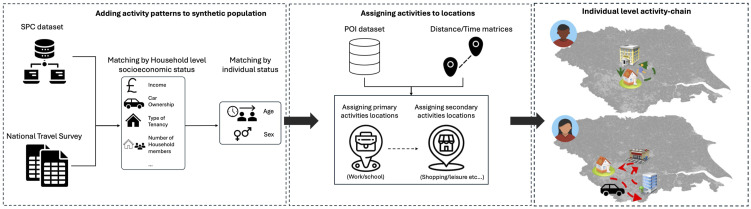
Process of creating an activity-based travel demand dataset, including assignment of (a) activity schedules and (b) activity locations.

**Figure 2.**
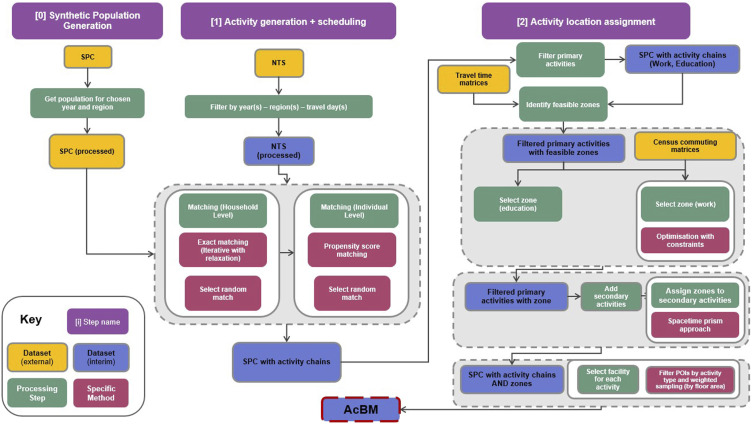
Detailed workflow of activity-based travel demand generation pipeline.

To run the pipeline, the datasets in Table 1 are used. A simplified overview of the different parts of the pipeline is in Figure 1. For a more detailed diagram of datasets and methodological steps, see Figure 2 in Appendix A.

**Table 1.** External datasets used in the pipeline.

Dataset	Purpose	Explanation
Synthetic Population Catalyst (SPC)	Core input	Synthetic population of England. All individuals have demographic characteristics, household composition, and home locations
National Travel Survey (NTS)	Generating activity schedules	Daily activity schedules for a sample of the population (trip sequences, purposes, distances, and time spent at each activity) as well as their demographic characteristics.
Point of Interest (POI) data	Primary and secondary location assignment	Labelled POI data from OSM. Used to assign individual activities to relevant locations
Travel time matrices	Primary location assignment	Travel time matrices by different modes. Used to assign activities to zones based on reported travel time in NTS. Estimates are made in absence of data
Census commuting matrices	Primary location assignment	Origin-destination commuting patterns (OA or MSOA level). Assigned work activities should match the geographic distribution of this dataset (see section 3.3 for implementation).

### Generating activity schedules

The first step is to add activity schedules to our synthetic population. To do so, we adopt a two-stage matching approach to maintain trip dependencies at the household level.

Step 1 (household level): In this step, we assign each household in the SPC to a household from the NTS. We use the household-level variables in Table 2 for matching. For each household in the SPC, we attempt to match on all variables, and iteratively relax the matching by removing variables (going up the table) until we find at least 5 matches from the NTS (the first three variable indicate household composition, so they should not be removed). We then randomly choose a matched NTS household from its pool of matches. The logic is described in Algorithm 1.

**Table 2.** Variables used for matching SPC to the NTS.

Variable	Type
No. of adults	Numerical
No. of children	Numerical
No. of pensioners	Numerical
No. of cars	Numerical
Rural/urban classification	Categorical (nominal)
Employment status	Categorical (ordinal)
Household income	Categorical (ordinal)
Type of tenancy	Categorical (nominal)

Step 2 (individual level): At this level, we are matching each individual to an activity schedule from the NTS. Each SPC household has a matched NTS household with the same number of people. To match at the individual level, we use an unconstrained statistical matching algorithm (Namazi-Rad et al., 2017). A nearest neighbour search is done to match individuals in the two datasets based on sex and age category. The matching is done iteratively and without replacement to ensure that all individuals in an SPC household are matched to unique individuals in the corresponding NTS household.


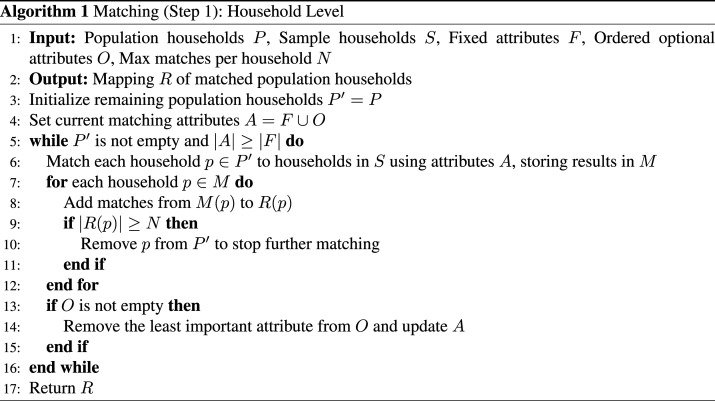


### Primary location assignment

Our approach to location assignment is to first assign people to home-based primary activity locations (work and education), and then to use a space-time prism approach for secondary activity assignment. This involves (a) determining, for each primary activity, the set of feasible zones where this activity could take place, and then (b) selecting a zone from the feasible zones. The detailed steps are as follows:

(1) Calculate a travel time matrix, by mode of transport. This can be done at OA or MSOA level. Ideally, a travel time matrix is calculated using a routing engine, and then used in our workflow. In the absence of a pre-calculated matrix, we calculate estimates in our workflow based on Euclidean distance and average travel speeds by mode. The estimated travel times are then adjusted to account for the difference between Euclidean and network distances. Following Prédhumeau and Manley (2025), we use Minkowski distance with coefficient (*λ*) of 1.56, but add a decay factor (*δ*) as discrepancy between Euclidean and network distances decreases as travel length increases (see equation 1). These factors are configurable and should be calibrated to each study area.
(2) Compare the reported travel time and mode of an activity to the travel time matrix and identify all destination zones that could be reached within a threshold percentage of the reported time (if reported time = 30 mins and our threshold is 20%, then all zones reachable within 30 ± 0.2 are shortlisted)
(3) Select a zone probabilistically based on the total area of the relevant facilities. For education trips, depending on the person’s age, we look at one of kindergartens, schools, and universities.
(4) Select a suitable facility in that zone.

(1) $$d_{\text{network}}=d_{\text{euclidean}}\cdot \left(1+\left(\lambda -1\right)\cdot e^{-\delta \cdot d_{\text{euclidean}}}\right)$$

**Figure 3.**
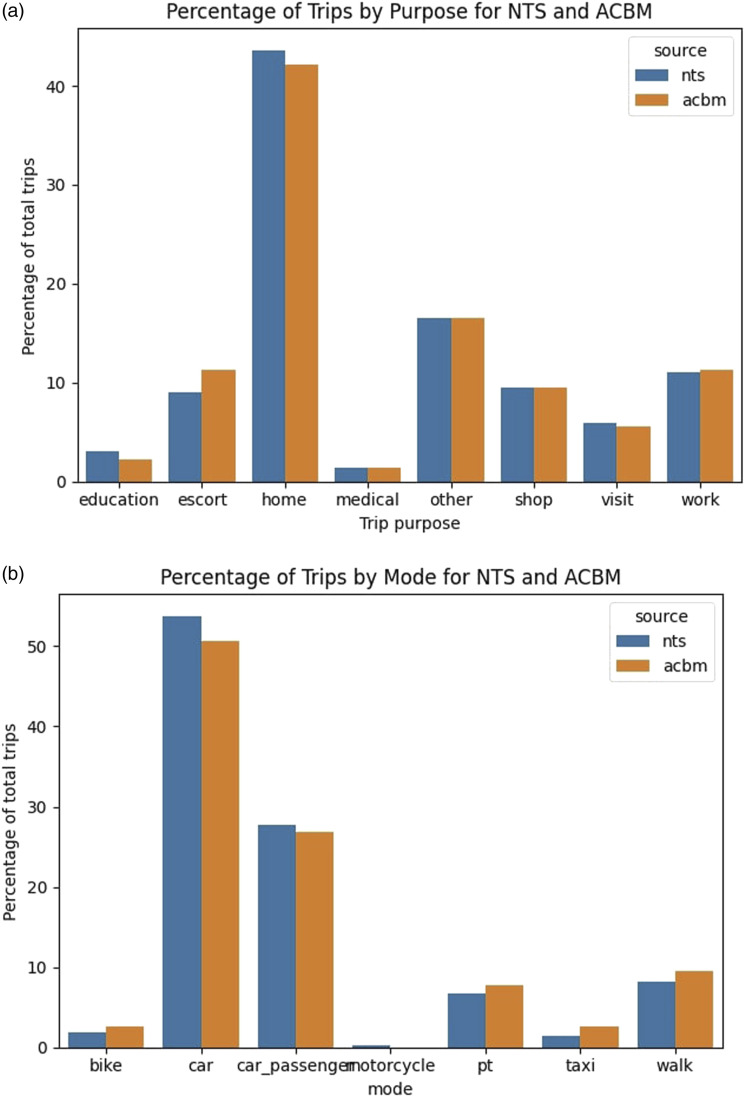
Comparing trip purpose and mode distributions between the NTS and our Activity-based model. (a) Trip purpose comparison; (b) Trip mode comparison

**Figure 4.**
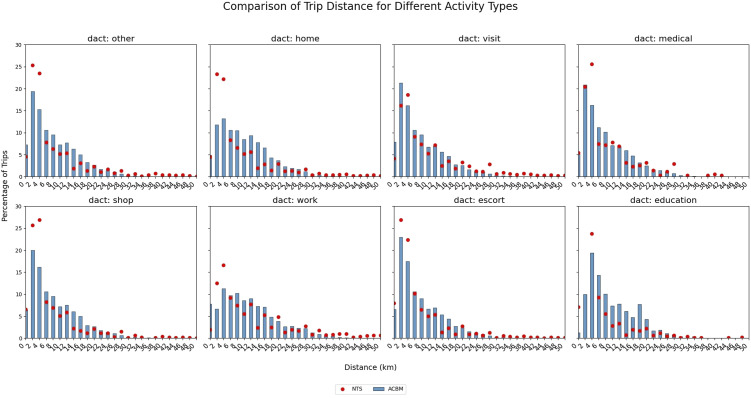
Trip length distribution: NTS versus AcBM. Secondary activities are assigned using an open-source solver (Shone et al., 2024) which assigns secondary activities in a sequence iteratively (see Section 3.4). (a) Trip purpose comparison. (b) Trip mode comparison.

The assignment of work locations is handled slightly differently from education locations. This is because a high-fidelity, ground-truth dataset for commuting is available in the form of the UK Census origin-destination (OD) matrices. Accurately reproducing these aggregate commuting patterns is important; work trips are typically longer than education trips (Figure 3 and DfT (2019)) and account for a higher proportion of total travel (Figure 4).

Therefore, using the reference census OD commuting matrices data, *we replace step 3 with an optimization problem* aimed at minimizing the divergence between our assignment and the census data. The problem is formulated as follows.

#### Variables

• *x*_*iod*_: Binary variable indicating whether individual *i* from origin zone *o* is assigned to destination zone *d* (1 if assigned and 0 otherwise).

#### Parameters

• *F*_*od*_: The actual flow (number of individuals) from origin zone *o* to destination zone *d*. This is obtained from a reference dataset (in this case the census commuting matrices).
• *T*_*o*_: The total flow for origin zone *o*, calculated as the sum of all flows originating from *o*:

(2) $$T_{o}=\underset{d}{\sum }F_{od}$$

• *Z*_*od*_: Binary parameter indicating whether destination zone *d* is feasible for origin zone *o* (1 if feasible and 0 otherwise).
• *α*, *β*: Weights for the two objectives.

#### Objective function

Minimize the weighted sum of:

(1) The sum of deviations between the assigned and actual flows (or percentages if using percentages).
(2) The maximum deviation across all OD pairs.

(3) $$\min\left(\alpha \underset{o,d}{\sum }\left|\frac{\sum_{i}x_{\mathrm{iod}}}{N_{o}}-\frac{F_{od}}{N_{o}}\right|+\beta \max_{o,d}\left|\frac{\sum_{i}x_{\mathrm{iod}}}{N_{o}}-\frac{F_{od}}{N_{o}}\right|\right)$$

where *N*_*o*_ is a normalization factor that adjusts the scale of the deviations depending on whether absolute flows or percentages are used:

(4) $$N_{o}=\left\{\begin{array}{cc} T_{o},\; & \text{if use\_percentages is True}\\ 1,\; & \text{otherwise} \end{array}\right.$$

If *use_percentages* is **True**, the deviations are computed in terms of percentages, where the percentage of flow from origin zone *o* to destination zone *d* is given by *F*_*od*_/*T*_*o*_. In this case, *N*_*o*_ is set to *T*_*o*_ so that both terms in the objective function are expressed as percentages. If *use_percentages* is **False**, the deviations are computed in absolute terms, and *N*_*o*_ is set to 1, keeping the objective function in terms of raw flow counts.

#### Constraints

(1) Flow conservation: Each individual must be assigned to exactly one destination zone:

(5) $$\underset{d}{\sum }x_{\mathrm{iod}}=1\;\forall i,o$$

(2) Non-negativity: The assignment variable *x*_*iod*_ should be binary (0 or 1):

(6) $$x_{\mathrm{iod}}\in \left\{0,1\right\}\;\forall i,o,d$$

(3) Feasibility: Individuals can only be assigned to feasible destination zones:

(7) $$x_{\mathrm{iod}}\leq Z_{od}\;\forall i,o,d$$

### Secondary location assignment

After assigning each individual to primary activity locations, we are left with secondary locations. We assign these activities to locations using a space-time prism approach, using the solver in the PAM library (Shone et al., 2024). The solver selects zones based on a combination of three metrics: leg ratio (travel time from previous activity/travel time to next activity.Compare reported ratio to candidate solutions), diversion factor (deviation from straight line between ‘anchor’ primary activities), and zone attraction (based on number of facilities).

## Pipeline configuration and consistency checks

The outputs of this pipeline are assessed using a series of internal consistency checks. A key challenge in validating a national-scale, general-purpose pipeline is that a validation performed for a single study area would not necessarily generalize to others, as the optimal parameters for one region may not be suitable for another. The purpose of the following checks is therefore to ensure the pipeline is functioning correctly and that key distributions within the input data are being plausibly reproduced.

Crucially, to facilitate external validation and calibration by the end user, the pipeline is designed to be configurable via a configuration file. Table 3 details a selection of parameters that a user can modify to adapt the pipeline’s behaviour across its major stages. This architecture allows users to tune the model to better match their specific local conditions or external datasets. For a complete and up-to-date guide to all parameters, users are directed to the documentation in the code repository.

**Table 3.** Examples of key configurable parameters in the pipeline.

Pipeline stage	Parameter	Purpose
Activity scheduling	*nts_region*	Selects appropriate National Travel Survey (NTS) regions to best represent the travel patterns of the study area
	*required_columns*, *optional_columns*	Defines the set of demographic variables used for stricter or more relaxed matching to NTS data
Primary location	*tolerance_work*, *tolerance_edu*	Sets the travel time tolerance (e.g. +/− 30%) for identifying feasible work or education zones from a person’s home location
	*detour_factor*, *decay_rate*	Calibrates the estimation of network travel distance from Euclidean distance. Edits allow adaptability to different street network topographies
	*weight_max_dev*, *weight_total_dev*	Adjusts the relative weights on (a) minimizing the maximum deviation, and (b) minimizing the total deviation in the work assignment optimization problem
Secondary location	*visit_probability_power*	Sets the distance-decay exponent in the gravity-based model for selecting discretionary locations. It controls how quickly the probability of visiting a zone decreases with increased travel distance, allowing calibration against observed trip length distributions

### Consistency check against NTS distributions

**Figure 5.**
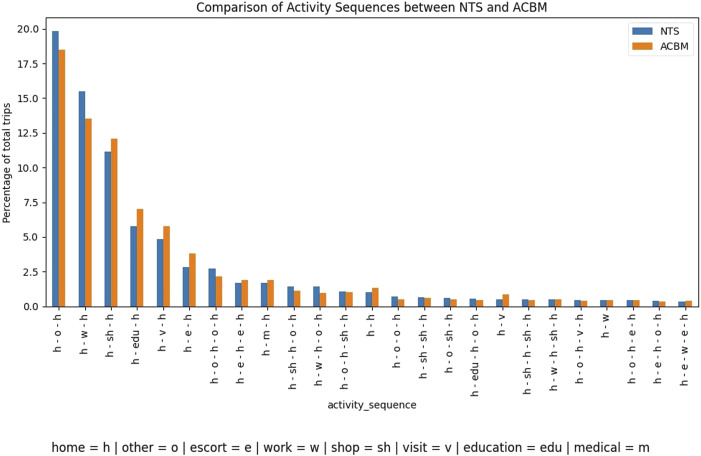
Comparison of most common activity sequence patterns between NTS and the activity-based model.

By comparing the pipeline’s output to the NTS, we can check that the pipeline plausibly reproduces the distributions from the survey data used as input. We include comparison plots for key travel characteristics, including mode shares, trip purposes, time of day by activity, travel distance by activity, and common activity sequences. Examples are shown in Figure 3, 4 and 5 in Appendix B.

### Consistency check against census commuting flows

The commuting flows are used as a constraint in our primary location assignment optimization problem. To assess how well the assignment matches this input data, we provide some goodness-of-fit statistics (R^2^, MAE, and RMSE).

**Figure 6.**
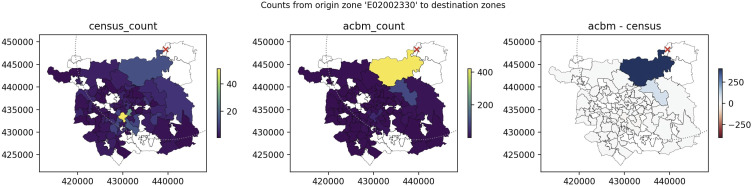
Counts for origin-destination flows for example boundary zone.

**Figure 7.**
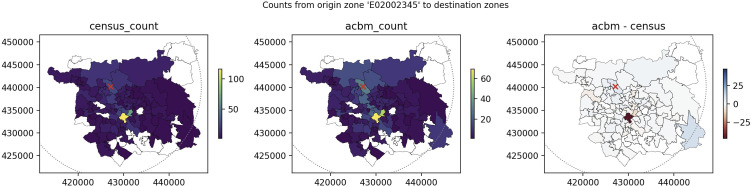
Counts for origin-destination flows for example centre zone.

In addition to these global measures, the pipeline also generates spatial diagnostic plots to help users assess model performance at a local level (equations (8) and (9)). These visualizations (shown in Figure 6 and 7 in Appendix C), which leverage methods from QUANT (Batty and Milton, 2021), allow for the identification of spatial patterns where the model may over- or under-predict travel flows. We provide these detailed diagnostics not as a final validation, but as a tool to aid users in their own calibration efforts. For example, a user could inspect these maps to guide the tuning of configurable parameters, such as the optimization weights, to improve the model’s fit for their specific study area.

Absolute map flow differences:

(8) $$G_{o}=\underset{d}{\sum }\left|T_{od}-F_{od}\right|$$

Percentage differences in local flow:

(9) $$q_{o}=\underset{d}{\sum }\left(\frac{T_{od}-F_{od}}{\sum_{o,d}F_{od}}\right)$$

where

*o*, *d* are indices representing all origin and destination zones in the system.
*T*_*od*_, *F*_*od*_ are the predicted and observed (from census data) number of commuters for a specific origin-destination pair (*o*, *d*).
*∑*_*o*,*d*_ *F*_*od*_ is the sum of all observed trips across all origin-destination pairs, representing the total flow in the entire system.
*G*_*o*_ calculates the total misallocation error for a specific origin zone *o*, representing the number of commuters assigned to an incorrect destination.
*q*_*o*_ calculates the net production error for a specific origin zone *o*. A positive value indicates the model has more total trips from that origin than observed, while a negative value indicates it has too few.

## Conclusion and future work

In this paper, we presented an activity-based travel demand generation pipeline that generates complete daily schedules and locations for synthetic populations. This open-source pipeline aims to reduce the time researchers spend on data preparation by providing a ready-to-use framework for generating the necessary input datasets for agent-based simulations. By automating simplifying the creation of activity-based travel demand datasets, the pipeline allows researchers to focus more on simulation and analysis, rather than dataset construction. Detailed documentation is available in the code repository*, allowing users to run the pipeline for any region in England. While the pipeline intentionally uses more established methods, the modular design also enables future extensions, such as integrating alternative methods for activity generation (Joubert and De Waal, 2020; Shone and Hillel, 2025), as well as refining primary (Zachos et al., 2024) and secondary (Hörl and Axhausen, 2023) location assignment.

## Data Availability

All the data sources used throughout this paper are publicly available and can be found as referenced in the text. The only exception is the National Travel Survey, which requires creating an account with the UK data service. We have put instructions here: https://github.com/Urban-Analytics-Technology-Platform/acbm/tree/main/data/external#data-sources. The code repository can be found at: https://github.com/Urban-Analytics-Technology-Platform/acbm/tree/main.

## Appendix

### Appemdix B. Self-consistency: Comparison to NTS

The figures below represent consistency checks against the NTS. Similar plots can be found in other papers that produce an activity-based travel demand dataset (Hörl and Balac, 2021; Prédhumeau and Manley, 2025; Sallard and Balać, 2023). These plots, as well those for the appendices below, are for a case study region of Leeds, UK.

#### Appendix C. Self-consistency: Comparison between census and AcBM commuting flows

The figures below show examples of comparing the distribution of commuting flows originating from a specific origin zone (marked by a red cross). For any origin, we can visualize the discrepancy between the distribution in the census data and the AcBM output. We show the census distribution (left), the AcBM distribution (centre), and the discrepancy between both (right).
